# Xanthones from the Pericarp of *Garcinia mangostana*

**DOI:** 10.3390/molecules22050683

**Published:** 2017-04-25

**Authors:** Renyue Yang, Ping Li, Nana Li, Qian Zhang, Xue Bai, Lishuo Wang, Yiying Xiao, Lirong Sun, Quan Yang, Jian Yan

**Affiliations:** 1 Key Laboratory of Tropical Agro Environment, Ministry of Agriculture and Guangdong Engineering Research Centre for Modern Eco-Agriculture, South China Agricultural University, Guangzhou 510642, China; renyueyang123@126.com (R.Y.); liping2016@scau.edu.cn (P.L.); lnnaxtj@163.com (N.L.); chermmon@163.com (Q.Z.); baixue0816@gmail.com (X.B.); wulidamonpalm@yahoo.com (L.W.); xyy15966@126.com (Y.X.); 2 Department of Neurobiology, School of Basic Medical Sciences, Southern Medical University, Guangzhou 510515, China; 3 Laboratory of State Administration of Traditional Chinese Medicine for Production and Development of Cantonese Medicinal Materials, School of Chinese Materia Medica, Guangdong Pharmaceutical University, Guangzhou 510006, China

**Keywords:** *Garcinia mangostana*, xanthone, cell lines, cytotoxic

## Abstract

Mangosteen (*Garcinia mangostana* L.) is one of the most popular tropical fruits (called the “Queen of Fruits”), and is a rich source of oxygenated and prenylated xanthone derivatives. In the present work, phytochemical investigation has resulted in one new prenylated xanthone and 13 known xanthones isolated from the pericarp of *G. mangostana*. Their structures were established by spectroscopic data analysis, including X-ray diffraction. The new one was further tested for cytotoxic activity against seven cancer cell lines (CNE-1, CNE-2, A549, H490, PC-3, SGC-7901, U87), displaying the half maximal inhibitory concentration (IC_50_) values 3.35, 4.01, 4.84, 7.84, 6.21, 8.09, and 6.39 μM, respectively. It is noteworthy that the new compound can promote CNE-2 cells apoptosis in late stage, having a remarkable inhibition effect on the side population growth of CNE-2 at 1.26 μM. The bioactive compound was also detected in extract from fresh mangosteen flesh, which indicated that the popular fruit could have potential cytotoxic activity for cancer cell lines.

## 1. Introduction

The genus *Garcinia* belongs to the Clusiaceae family, and a total of 21 species of the genus have been recorded to date in China [1]. *Garcinia mangostana* (mangosteen) is a tropical fruit native to the Malay Archipelago, the Sunda Islands, and the Moluccas. In Southeast Asia, mangosteen fruit shell is a traditional folk medicine used for the treatment of diarrhea, sprains, typhoid, ulcers, skin infections, and is used as an anti-inflammatory and for sterilization. In the previous extensive investigations on the phytochemical constituents of *G. mangostana* [2], *G. hanburyi* [3], *G. bracteata* [4], *G. cowa* [5], etc., *Garcinia* species are known to be rich in a variety of benzophenones and xanthones, some of which showed a wide range of biological and pharmacological activities including cytotoxic, anti-inflammatory, antimicrobial, and antifungal activity, as well as immune regulation and the amelioration of experimental autoimmune encephalomyelitis [6,7,8,9,10,11,12,13]. A dietary α-mangostin isolated from genus *Garcinia* had anticancer and antiproliferative properties in leukemia as well as prostate, breast, colorectal, and brain cancers [14], and another caged polyprenylated xanthone—gambogic acid—was exhibited to have anti-proliferative and pro-apoptotic effects on hepatocarcinoma, gastric carcinoma, lung carcinoma, breast cancer, and glioma in vivo and in vitro [15].

In China, *G. mangostana*—also named as Shan Zhu, mountain bamboo imported from Thailand, Malaysia and other Southeast Asian countries—was one of the most popular fruits, called the “Queen of Fruits” for its sweetness and juiciness as well as its importance in enhancing a person’s health [16]. In recent years, increasing evidence has supported that diet plays an important role in preventing the development of cancer [8,17]. Fruits are one of the main dietary components for daily consumption. It is popularly believed that increasing fruit consumption will contribute to reduced risk of cancers of oral cavity, pharynx, larynx, esophagus, stomach and lung [18] due to the intake of some specific active substances as we consume fruit every day.

In the course of our scanning for bioactive constituents from mangosteen fruits, one new prenylated xanthone named as 7-*O*-demethyl mangostanin (**1**) and 13 known xanthones (**2**–**14**) were isolated from the pericarp, and their structures were established using nuclear magnetic resonance (NMR), high resolution electrospray ionization mass spectroscopy (HRESIMS), and X-ray methods. Compound **1** showed obvious inhibition activity of all tested cancer cell lines, and the content of compound **1** in edible fruit flesh is 13.89 ± 0.57 μg/kg, suggesting that the fruit could be of potential value for the prevention and treatment of cancer.

## 2. Results and Discussion

**Figure 1:**
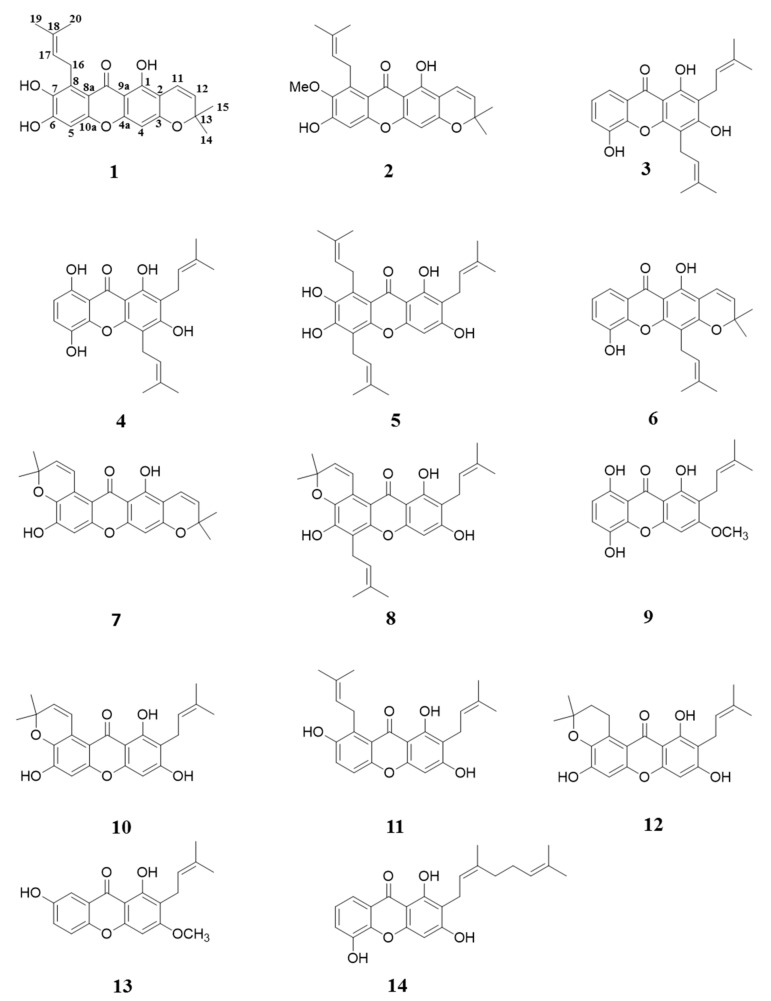
Chemical structure of xanthones **1**–**14** isolated from *Garcinia mangostana* L.

Repeated column chromatography of 95% ethanol crude extraction of *G. mangostana* led to the isolation of one new compound (**1**), and 13 known compounds (Figure 1). The chemical structures of known compounds mangostanin (**2**) [19], 8-deoxygartanin (**3**) [20], gartanin (**4**) [21], garcinone E (**5**) [22], trapezifolixanthone (**6**) [23], padiaxanthone (**7**) [7], tovophyllin A (**8**) [24], 1,5,8-trihydroxy-3-methoxy-2[3-methyl-2-butenyl] xanthone (**9**) [25], garcinone B (**10**) [6,26], 1,3,7-trihydroxy-2,8-di-(3-methylbut-2-enyl)xanthone (**11**) [27], mangostenone D (**12**) [6], 2-geranyl-1,3,5-trihydroxyxanthone (mangostinone) (**13**) [28], and 1,7-dihydroxy-2-(3-methylbut-2-enyl)-3-methoxyxanthone (**14**) [29] were identified by comparing spectroscopic data with those of published values. The chemical structure of the new compound was elucidated as follows.

**Figure 2:**
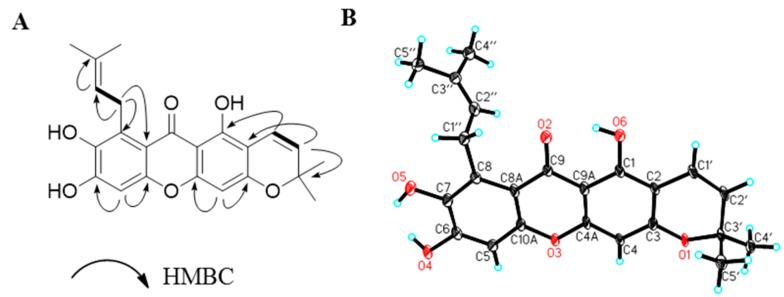
Identification of chemical structure of compound **1** from *G. mangostana*. (**A**) Selected heteronuclear multiple bond correlation (HMBC) correlations (H → C) of **1** and (**B**) X-ray crystallographic analysis of **1**.

7-*O*-Demethyl mangostanin (**1**) was obtained as a yellow solid. Its molecular formula was determined to be C_23_H_22_O_6_ by the quasi-molecular ion peak [M + H]^+^ at *m*/*z* 395.1507 in HRESIMS. The ^1^H and ^13^C-NMR spectra showed some characteristic signals of tetracyclic xanthone with a 3-methylbut-2-enyl-group and a pyrano ring (Table 1). There is a chelated hydroxyl group at δ_H_ 14.08 (1H, s, 1-OH), a chelated carbonyl at δ_C_ 181.7 (s, C-9), two characteristic proton and carbon signals of the xanthone [δ_H_ 6.32 (1H, s, H-4), 6.76 (1H, s, H-5) and δ_C_ 93.6 (d, C-4), 100.1 (d, C-5)], two coupled aromatic protons [δ_H_ 6.61 (1H, d, 9.9, H-11) and 5.73 (1H, d, 9.9, H-12), δ_C_ 114.8 (d, C-11), 128.0 (d, C-12)], and a 3-methylbut-2-enyl-group signal [δ_H_ 4.02 (2H, d, 6.8, H-16), 5.18 (1H, t, H-17), 1.61 (3H, s, H-19) and 1.78 (3H, s, H-20); δ_C_ 25.3 (t, C-16), 123.5 (d, C-17), 130.2 (s, C-18), 25.67 (t, C-19) and 18.3 (t, C-20)] in the NMR spectra. Comparing NMR data of **1** with mangostanin (19), the chemical shift values at C-6 and C-7 was moved to up-field due to existence a chelated hydroxyl group, and two significant broad hydroxyl signals at δ_H_ 11.2 (1H, OH-7) and 8.7 (1H, OH-8) were observed in the ^1^H NMR. The assignments of protonated and quaternary carbons were assigned from heteronuclear singular quantum correlation (HSQC) and heteronuclear multiple bond correlation (HMBC) spectra (see Table 1). The connection of the 3-methylbut-2-enyl moieties at C-8 was established on the basis of the HMBC correlations of H-16 to C-8 and C-7. Two aromatic protons were assigned to positions C-4 and C-5 from the HMBC correlations of H-4 at δ_H_ 6.32 (1H, s) with C-2 (δ_C_ 103.6), C-32 (δ_C_ 158.4), and C-9a (δ_C_ 102.9) and correlations of H-5 at δ_H_ 6.32 (1H, s) with C-6 (δ_C_ 152.8), C-7 (δ_C_ 141.2), C-8a (δ_C_ 109.6), and C-10a (δ_C_ 152.0) (see Figure 2A). The structure was further confirmed by X-ray diffraction (see Figure 2B). Compound **1** was therefore identified as 1,6,7-trihydroxy-8-isoprenyl-6′,6′-dimethylpyrano (2,3′:3,2) xanthone, named as 7-*O*-demethyl mangostanin.

In the activity evaluation of xanthones from the pericarp of *G. mangostana*, compounds **1**–**14** were tested for their inhibition effect on two common cell lines using 3-(4,5-dimethyl-2-thiazolyl)-2,5-diphenyl-2-*H*-tetrazolium bromide (MTT) method (see Table 2 and refer to Tables S1 and S2). Six compounds **1**, **2**, **3**, **4**, **5**, **11** inhibit PC12 (pheochromocytoma cell) and U87 (human malignant glioma cell) cell proliferation in a dose-dependent fashion, but the six compounds had stronger activities against U87 than PC12. By comparing their chemical structure–activity relationship, the activity was reduced with pyrano ring at C-7 and C-8 like compounds **7**, **8**, **10**, and **12**. The cytotoxicity was weaker with the methyl group at C-7 comparing **2** with **1**. To intensively assess the potential cytotoxic activity of the new compound **1**, **1** was tested by seven cell lines CNE-1, CNE-2 (nasopharyngeal carcinoma cell line), A549, H460 (lung cancer cell line), SGC-7901 (gastric cancer cell line), PC-3 (prostate cancer cell line), and U87, and the half maximal inhibitory concentration (IC_50_) values were 3.35, 4.01, 4.84, 7.84, 6.21, 8.09, and 6.39 μM, respectively (Table 2). Hirsutanol A was used as positive control, and the IC_50_ were 9.52, 11.86, 12.00, 11.00, 11.25, and 15.00, 6.3 μM. Compound **1** was greater than positive control Hirsutanol A.

**Figure 3:**
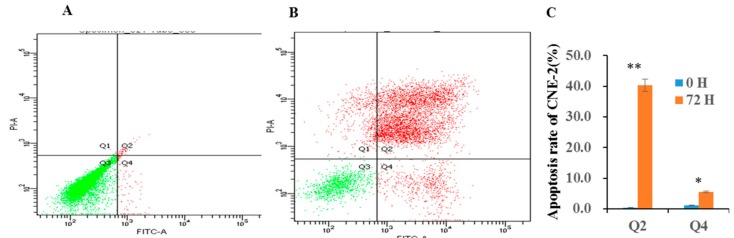
Compound **1** can promote CNE-2 cell apoptosis. Cells were labeled with Annexin-Fluorescein isothiocyanate (FITC) and Propidium Iodide (PI), and analyzed by flow cytometry (FCM): (**A**) untreated; (**B**) treated with 6.34 μM for 72 h; (**C**) Effects of compound **1** on apoptosis in CNE-2 cell. Q2 and Q4 stand for late stage and early stage, respectively. * *p* < 0.05, ** *p* < 0.01 compared with the control group. Data are expressed as the mean ± standard deviation (SD). *n* = 3.

**Figure 4:**
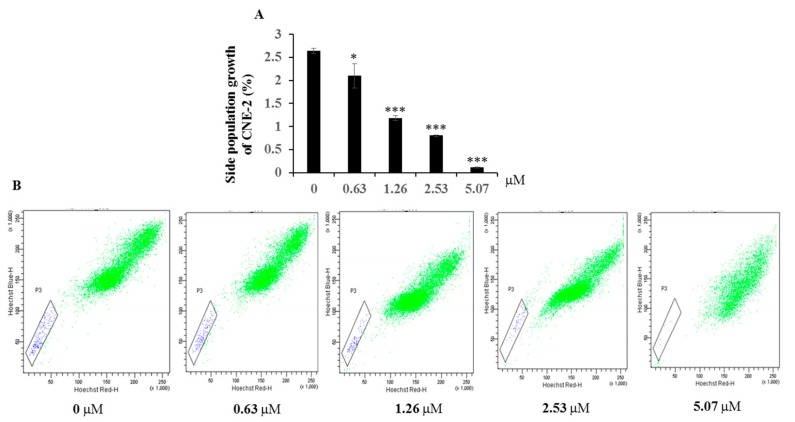
Compound **1** effect of side population growth CNE-2. (**A**) Compound **1** inhibited side population growth in a concentration-dependent manner as determined by FCM analysis. (**B**) The inhibition was visualized by adding Annexin-FITC and PI at different concentrations. * *p* < 0.05, *** *p* < 0.001 compared with the control group. Data are expressed as the mean ± SD. *n* = 3.

Programmed cell death mainly occurs through either type I cell death (apoptosis) or type II cell death (autophagy). The present results showed that compound **1** can significantly induce the late and early stage apoptosis of CNE-2 cell at 6.34 μM—40.3% and 5.5%, respectively (see Figure 3). Side population cells are commonly thought to represent cancer stem cell populations, and inhibition of the growth of cancer stem cell populations is an effective way to treat cancer. The inhibition effect of compound **1** on the side population of CNE-2 cell was measured by flow cytometry (FCM). It showed that compound **1** had very significant cytotoxic active against the growth of side population of CNE-2 cell from 1.26 to 5.07 μM (see Figure 4).

The content of bioactive compound **1** from fresh aril was 13.89 ± 0.57 μg/kg by ultra-performance liquid chromatography-electrospray ionization-tandem mass spectrometry (UPLC-ESI-MS/MS) analysis (Table S3). The new compound is not only isolated from pericarp, but is also distributed in flesh aril. The bioactive compound **1** could be potential matter for cytotoxic activity from fresh *G. mangostana*, suggesting that animal experiments and pharmacological analysis is worth carrying out to support the opinion.

## 3. Materials and Methods

### 3.1. General Experimental Procedures

NMR spectra were recorded on a Bruker advance 400 NMR spectrometer (Bruker Biospin, Zurich, Switzerland). HRESIMS mass spectra were obtained on an ultra-performance liquid chromatography (UPLC) 1290-6540B quadrupole-time of flight (Q-TOF) instrument (Waters. Ltd., Milford, MA, USA) in positive ion mode after direct injection of the test solutions. ESIMS data were obtained using a MDS SCIEX API 2000 LC/MS/MS system (Waters. Ltd., Milford, MA, USA). UPLC-MS was carried out on a C_18_ column (ACQUITY UPLC BEH C18 2.1 × 50 mm i.d., 1.7 μm, Waters. Ltd., Milford, MA, USA). UPLC-TQS-MS was operated using an Acquity UPLC system (Waters Corporation, Milford, MA, USA) coupled with a MS (Xevo TQ-S, Waters MS Technologies, Manchester, UK), controlled by MassLynx v4.1 software (Waters Corp., Milford, MA, USA). Open column chromatography (CC) was carried out using silica gel (80–100 and 200–300 mesh, Qingdao Haiyang Chemical Co. Ltd., Qingdao, China), and Sephadex LH-20 (Pharmacia Fine Chemical Co. Ltd., Uppsala, Sweden). Thin layer chromatography (TLC) was performed on HSGF254 TLC (Yantai Jiangyou silica gel Co. Ltd., Yantai, China), and spots were visualized by heating the silica gel plates sprayed with 10% sulphuric acid in ethanol (*v*/*v*). HPLC-MS-grade acetonitrile, water, and formic acid were purchased from J. T. Baker (Philipsburg, NJ, USA). All analytical-grade reagents were purchased from the Tianjin Fuyu Fine Chemical Industry Co. Ltd., Tianjin, China.

### 3.2. Plant Material

The fresh *G. mangostana* of Thailand was purchased from Guangzhou market in July 2015. A dry voucher specimen (No.: 20160105GM) has been deposited in the herbarium of the College of Natural Resources and Environment, South China Agricultural University, China.

### 3.3. Sample Preparation and Isolation

For the extraction and isolation of compounds from the pericarp of *G. mangostana*, the pericarps were separated from fresh fruits and naturally dried, then milled with a grinder, and 1 kg of material was extracted with 95% ethanol to obtain crude extract (188 g). Then, 150 g silica gel (80–100 mesh) was used to mix with crude extract (128 g) and was directly filled into the silica gel column chromatograph (70 × 920 mm, 200–300 mesh), eluted with petroleum ether, dichloromethane, and dichloromethane:methanol (50:1, 20:1, 10:1, 5:1), respectively. The collected fractions were monitored by TLC, and combined to yield five fractions (Fr.1–2). Fr.1 (52 g) was chromatographed on a silica gel column washed with petroleum ether–dichloromethane (10:1, 5:1, and 1:1, *v*/*v*) to obtain sub-fractions Fr.1.1, 1.2, and 1.3. Fr.1.1 was further repeatedly chromatographed on a silica column eluting with petroleum ether–dichloromethane (15:1, 7:1, 3:1, *v*/*v*) to get compounds **2** (13 mg), **3** (9 mg), **4** (20 mg), **5** (10 mg), and **6** (7 mg). Fr.1.2 was subjected to a silica column eluting with petroleum ether–dichloromethane (2:1 and 1:1, *v*/*v*) to yield compounds **2** (5 mg), **7** (7 mg), and **8** (11 mg). Fr.1.3 was subjected to a silica gel column eluting with gradient system chloroform–methanol (30:1, 20:1, and 10:1, *v*/*v*), and further purified by Sephadex LH-20 with eluting methanol to give **9** (20 mg), **10** (6 mg), and **11** (13 mg). The Fr.2 was rechromatographed on a silica column with chloroform–methanol (70:1, 60:1, and 50:1, *v*/*v*) as gradient system to obtain **1** (10 mg), **11** (10 mg), **12** (18 mg), **13** (8 mg), and **14** (11 mg).

7-*O*-demethyl mangostanin (**1**): yellow solid, IR ν max cm^−1^: 3500 (OH), 1650 (C=O), 1604 (Ar). ^1^H- and ^13^C-NMR (DMSO-*d*_6_) data, see Table 1; HRESIMS *m*/*z* 395.1507 [M + H]^+^ (C_23_H_23_O_6_ calcd for 395.1416).

Crystallographic data for **1**: C_23_H_22_O_6_·C_3_H_6_O, MW = 452.48, monoclinic, *a* = 6.1328(5) Å, *b* = 19.7854(13) Å, *c* = 18.1183(13) Å, α = 90.00°, β = 90.00°, γ = 90.00°, V = 2198.5(3) Å_3_, T = 100(2) K, space group P21/c, Z = 4, μ (CuKα) = 0.816 mm^−1^, 10,923 reflections measured, 3497 independent reflections (Rint = 0.1292). The final R_1_ values were 0.1121 (I > 2σ (I)). The final Wr (F_2_) values were 0.2686 (I > 2σ (I)). The final R_1_ values were 0.1670 (all data). The final wR (F_2_) values were 0.3160 (all data). The goodness of fit on F2 was 0.982.

For the determination of bioactive constituents from mangosteen, the fresh aril segments were carefully separated into arils (edible parts) and seeds by a stainless-steel knife. The fresh arils (each 4 g) were each stirred with 2 mL 95% ethanol followed by ultrasonic extraction for 30 min in a water bath at room temperature. After membrane-filtration (0.25 μm), the extract solutions were directly used for the quantification of bioactive compounds by UPLC-QTS-MS analysis.

UPLC-TQS-MS analysis: Quantitative analysis of samples was performed on an ACQUITY UPLC system couple with a triple-quadrupole Xevo TQ-S mass spectrometer (Waters Corp., Milford, MA, USA). An ACQUITY UPLC BEH C_18_ column (2.1 mm × 50 mm, 1.7 μm) (Waters Corp., Milford, MA, USA) was employed, and the column temperature was maintained at 40 °C. The gradient elution with acetonitrile containing 0.1% formic acid (A) and water containing 0.1% formic acid (B) was performed as follows: 0–0.5 min, 30–35% A; 0.5–2 min, 35–75% A; 2–3.5 min, 75–90% A; 3.5–4.5 min, 90–95% A; 4.5–4.8 min, 95–30% A; 4.8–6 min, 30% A. The flow rate was set at 0.4 mL/min. The auto-sampler was conditioned at 25 °C, and the injection volume of solution was 5 μL for analysis. Mass spectrometric detection was performed on Xevo TQ-S equipped with an electrospray ionization source (ESI). The capillary voltage was set to 3.5 kV, and the source temperature was maintained at 150 °C. The collision gas was Ar, and N_2_ gas was used as desolvation at temperature of 400 °C and cone gas at a flow rate of 700 L/h, the cone gas set to 50 L/h. Compound **1** was optimized in multiple reaction monitoring in positive mode. The cone voltage was 48 V, *m*/*z* 394.0 was selected as parent ion, and *m*/*z* 323.8 and 339.0 were set as the daughter qualitative and quantitative ions, respectively, with the corresponding collision energy settings at 38 and 28 V. The dwell time was 0.025 s. Targetlynx (Waters Corp., Milford, MA, USA) software was used to analyze the data.

### 3.4. Cell Culture

All cell lines were cultured in dulbecco’s modified eagle's medium (DMEM) medium containing 10% fetal bovine serum, 5% horse serum, 100 U/mL penicillin, and 100 mg/mL streptomycin. All cell lines were placed in an incubator at 37 °C with 5% carbon dioxide.

### 3.5. MTT Assay

Cells were seeded into 96-well plates (about 3000 cells/well), and were treated with seven concentration gradients: 0, 0.79, 1.58, 3.17, 6.34, 12.59, 25.38, 50.76, and 101.56 μM, then the cells were incubated for 48 h. Subsequently, MTT was added to the culture medium to yield a final MTT concentration of 800 μg/mL, and cells were incubated with the MTT for 4 h in the incubator, then collected and dissolved in dimethyl sulphoxide (DMSO). Colorimetric analysis was measured at 570 nm.

### 3.6. FCM Assay

Cells were treated with 6.34 μM compound **1**, then incubated for 72 h, cells were collected, and Annexin-FITC and PI were added; after dark reaction for 10 min, cell apoptosis was analyzed using FCM for the treated group and the control group.

## 4. Conclusions

In summary, 14 compounds were isolated and identified from *G. mangostana*. The new compound was tested by seven cancer cell lines and side population growth of CNE-2, showing that the compound has potential anti-cancer properties. It is noteworthy that this bioactive constituent can also be detected in mangosteen aril. There is some possibility that eating mangosteen is useful for the prevention and treatment cancer.

## Figures and Tables

**Table 1:** ^1^H- and ^13^C-NMR spectral data of compound **1** in DMSO-*d*_6_.

Position	Compound 1	
	^13^C	^1^H
1	157.1	
2	103.6	
3	158.4	
4	93.6	6.32, s
4a	155.6	
5	100.1	6.76, s
6	152.8	
7	141.2	
8	127.6	
8a	109.6	
9	181.7	
9a	102.9	
10a	152.0	
11	114.8	6.61, d, 9.9
12	128.0	5.73, d, 9.9
13	77.9	
14	27.4	1.43, s
15	27.4	1.43, s
16	25.3	4.02, d, 6.8
17	123.5	5.18, t, 6.8
18	130.2	
19	25.7	1.61, s
20	18.3	1.78, s

DMSO: dimethyl sulfoxide; δ in ppm, *J* in Hz.

**Table 2:** Cytotoxic activities of compound **1**.

Concentration (μM)	Cell Lines							
	CNE-1	CNE-2	A549	H460	PC-3	SGC-7901	U87	PC12
0	0	0	0	0	0	0	0	0
0.40	0	5.4	10.6	0	7.27	1.94	0	/
0.81	4.35	12.87	11.11	0	10.08	3.37	14.26	/
1.58	47.42	15.96	23.81	1.15	12.27	17.98	16.25	/
3.17	63.68	51.92	49.6	20.04	32.93	47.7	26.55	2.19
6.34	69.4	71.24	62.54	50.72	58.68	49.17	49.15	11.33
12.69	78.42	80.01	63.18	58.45	64.59	51.74	68.24	4.66
25.3	83.9	83.89	73.98	84.86	72.64	54.91	72.56	23.82
50.7	/	/	/	/	/	/	/	40.57
101.5	/	/	/	/	/	/	/	58.21
IC_50_	3.35	4.01	4.84	7.84	6.21	8.09	6.39	>50
							(2.51 μg/mL)	(19.7 μg/mL)

IC_50_: half maximal inhibitory concentration; /: no detection.

## Supplementary Materials

NMR data of **1**, Growth (%) of tumor PC12 and U87 and linear regression data of **1** are available online.
