# Supplementary material for: Xanthones from the Pericarp of Garcinia mangostana
Source: Molecules. 2017 Apr 25;22(5):683. doi: 10.3390/molecules22050683 (PMC6154529; doi:10.3390/molecules22050683)
Supplement: Supplementary file 1 [file molecules-22-00683-s001.pdf]

1HNMR spectrum of sample II-1-2

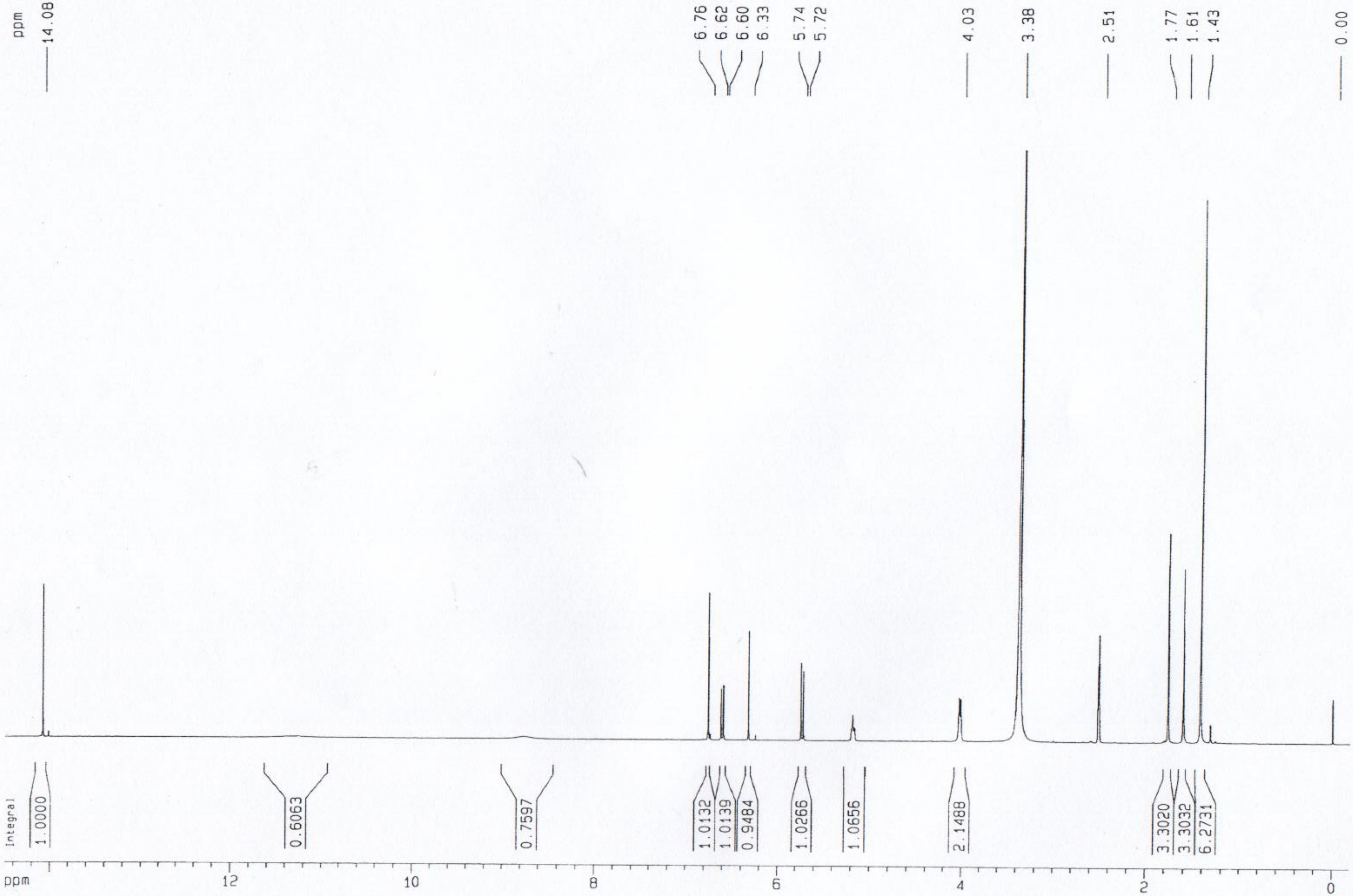

13CNMR spectrum of sample II-1-2

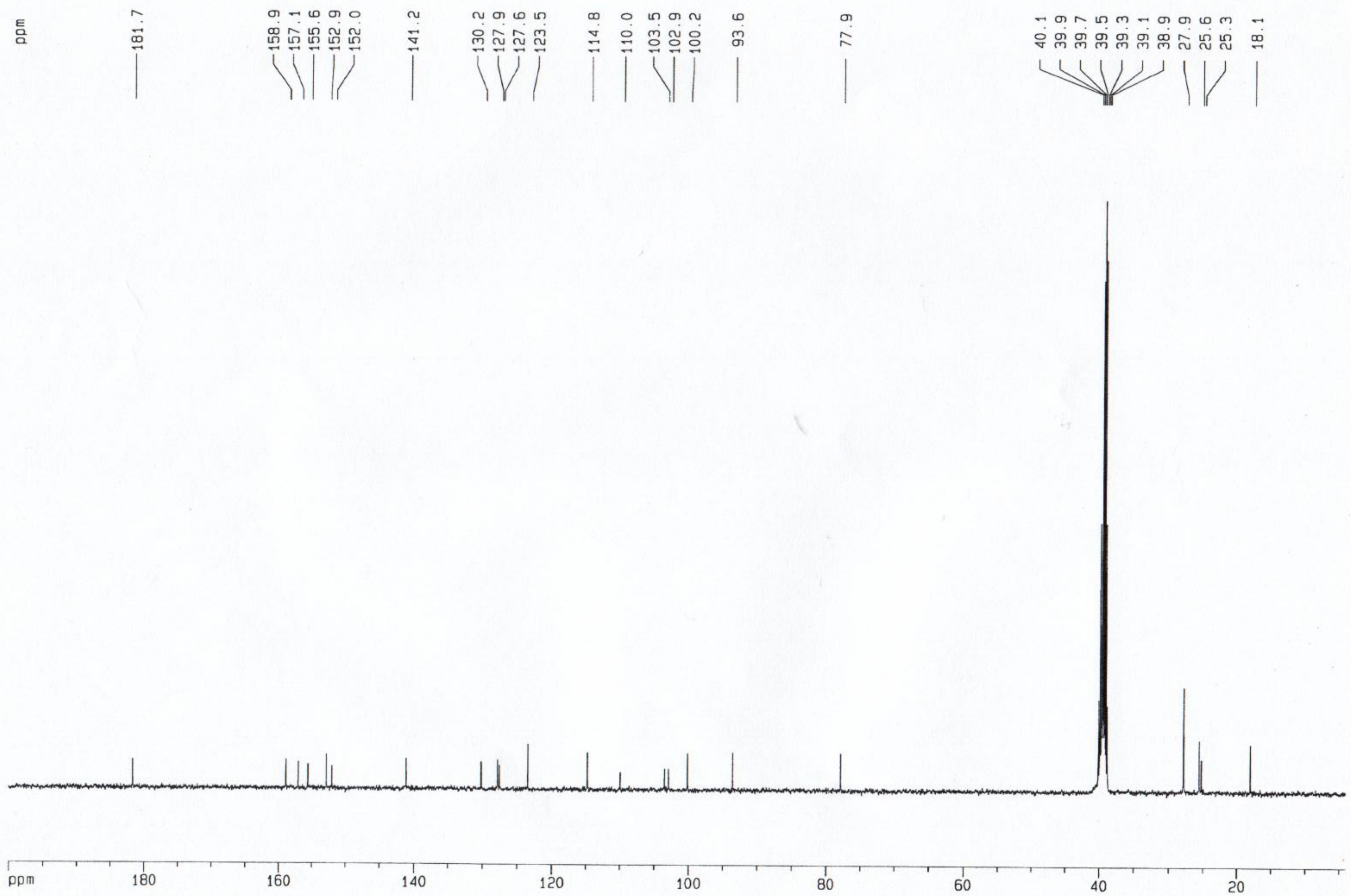

DEPT135 of sample II-1-2

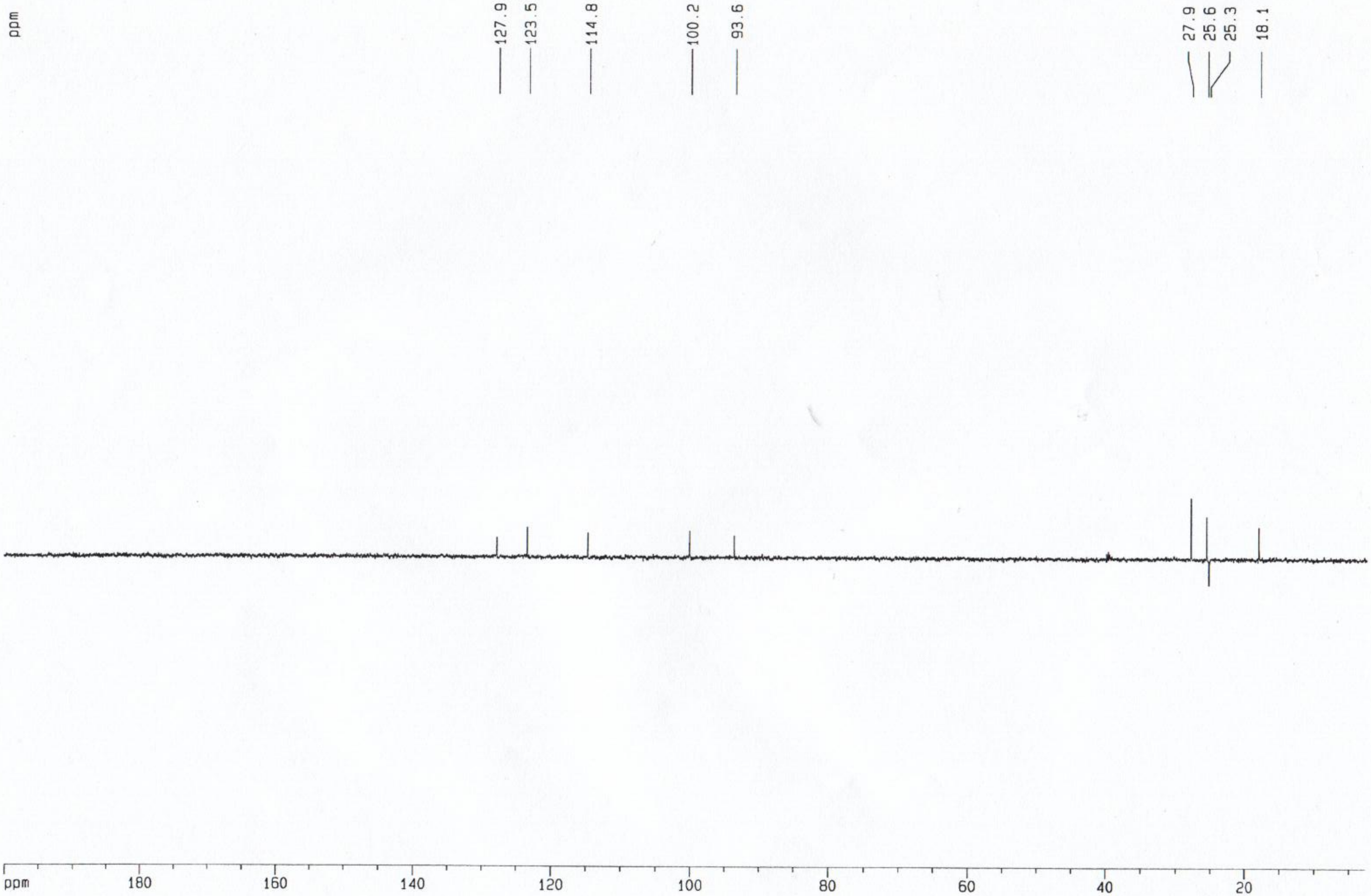

DEPT90 of sample II-1-2

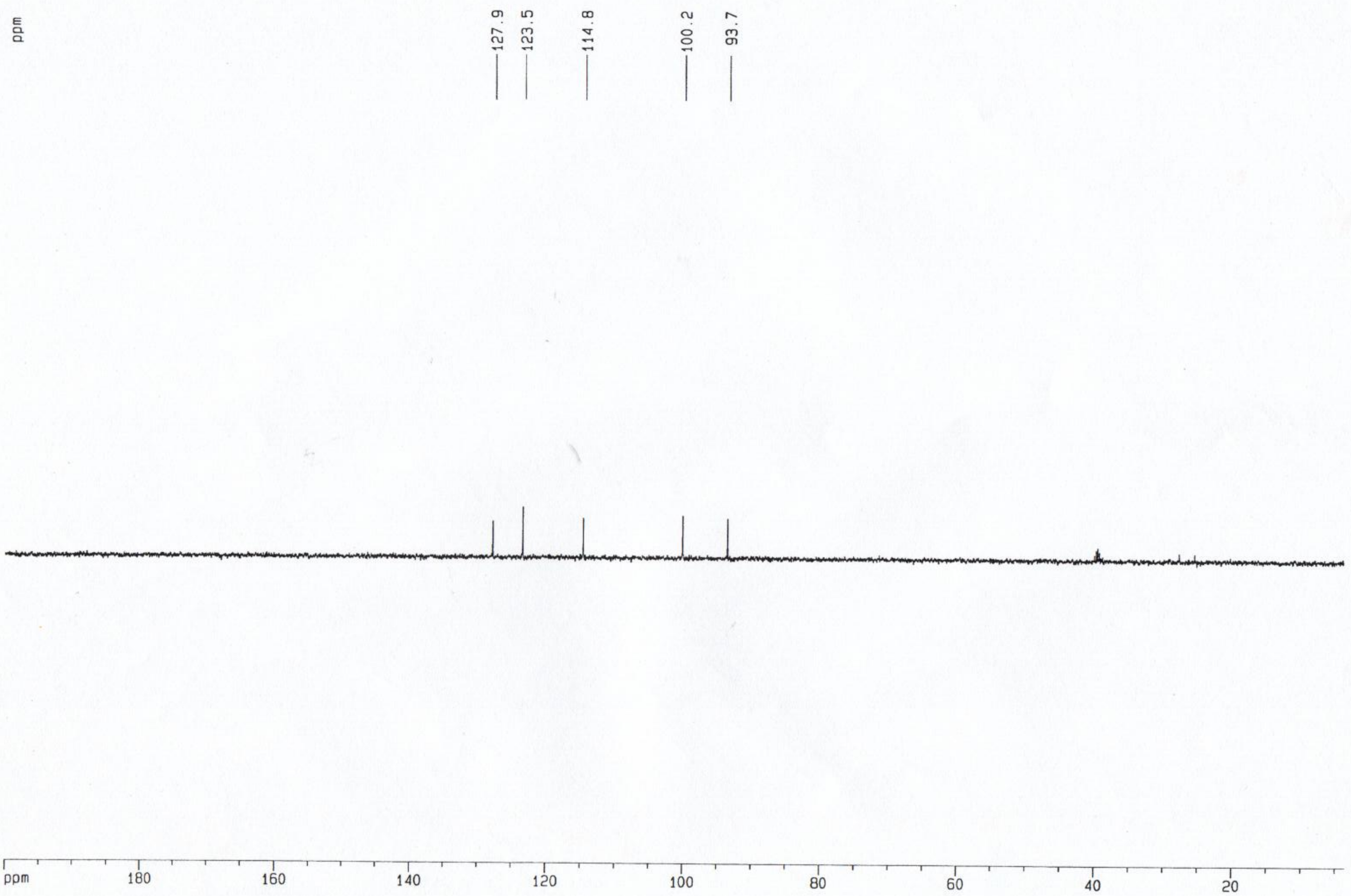

13C-1H COSY of sample
II-1-2

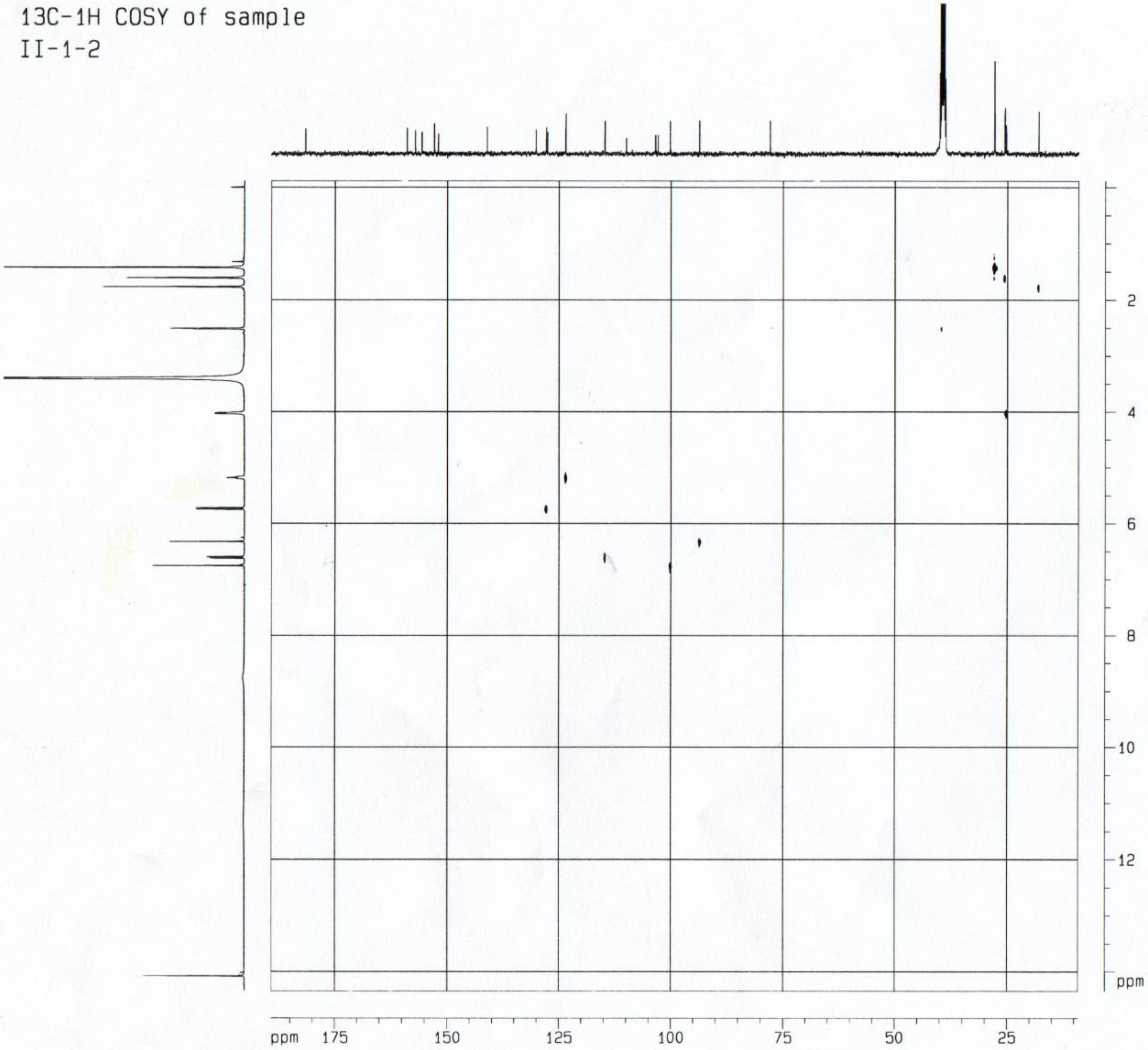

13C-1H COSY of sample
II-1-2

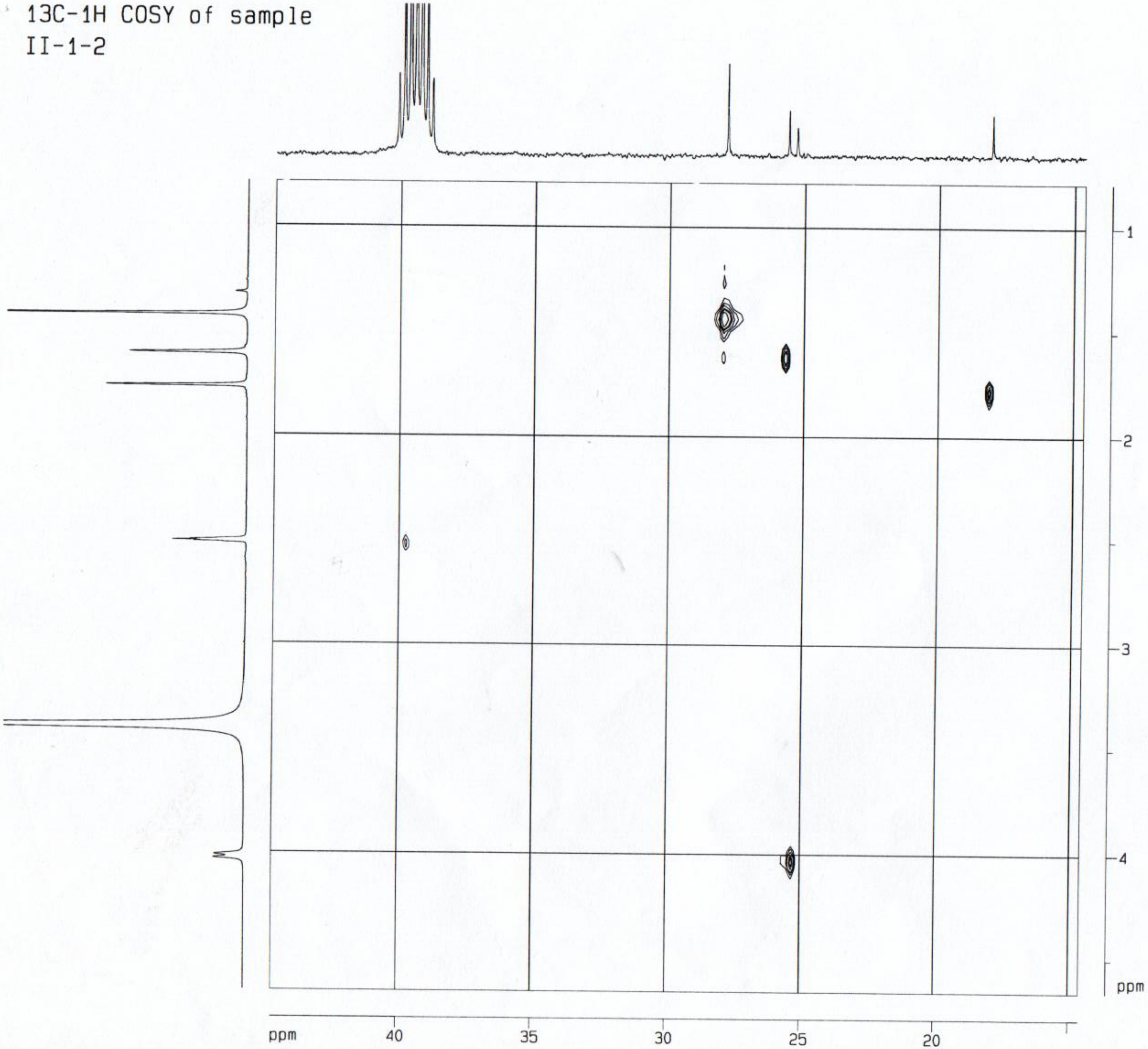

13C-1H COSY of sample
II-1-2

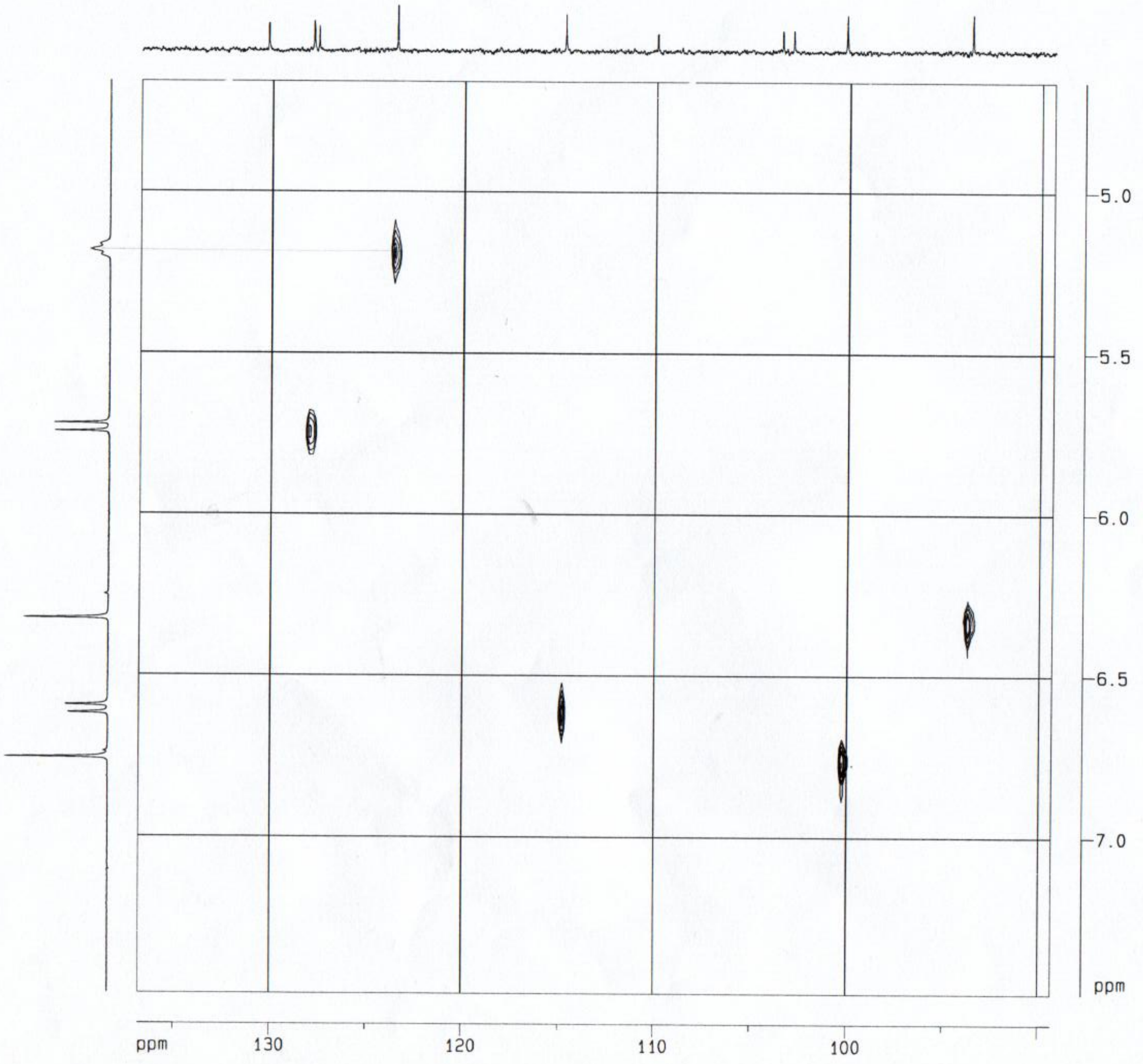

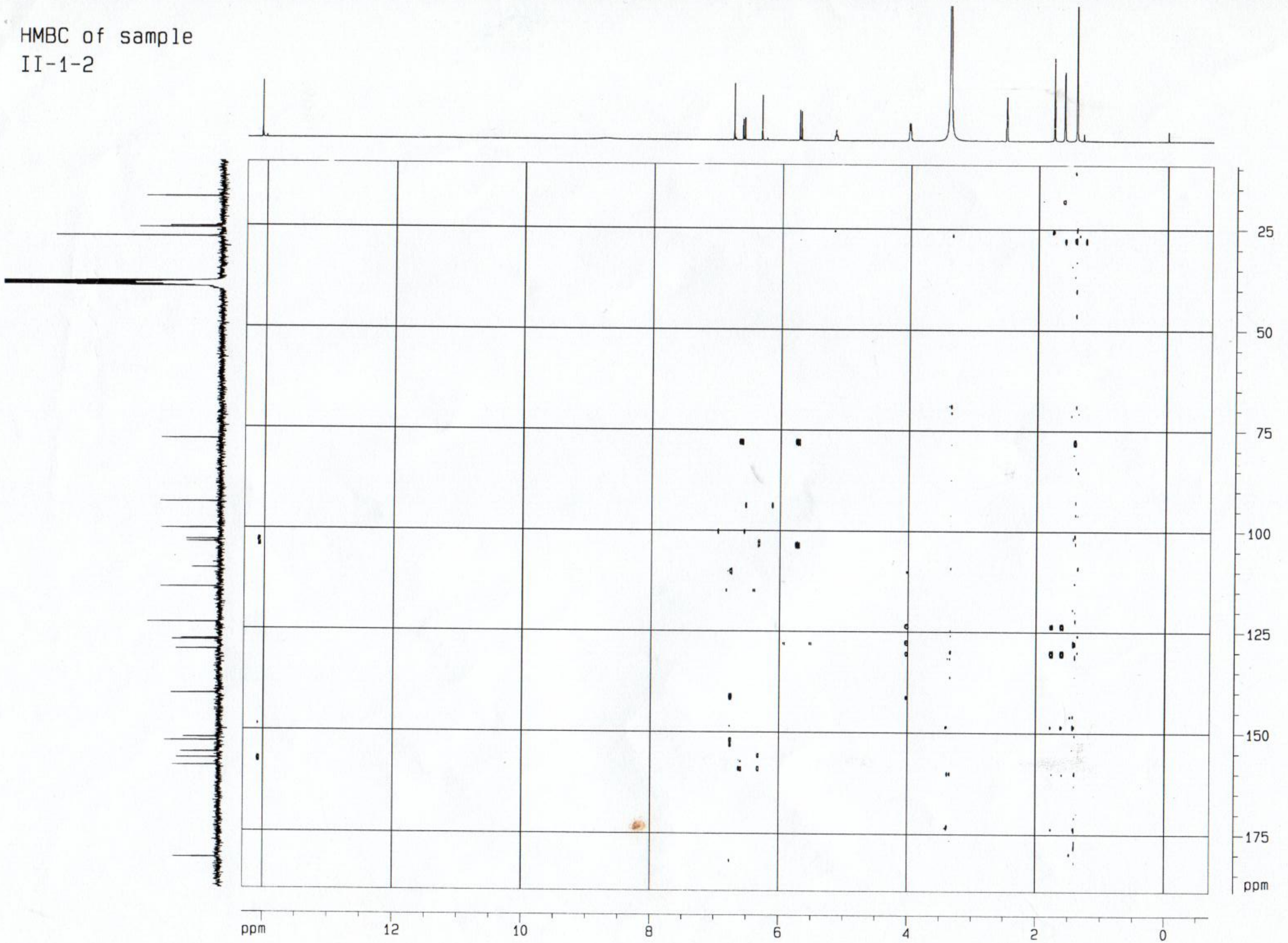
HMBC of sample
II-1-2
ppm
12
10
8
6
4
2
0
25
50
75
100
125
150
175
ppm

HMBC of sample
II-1-2

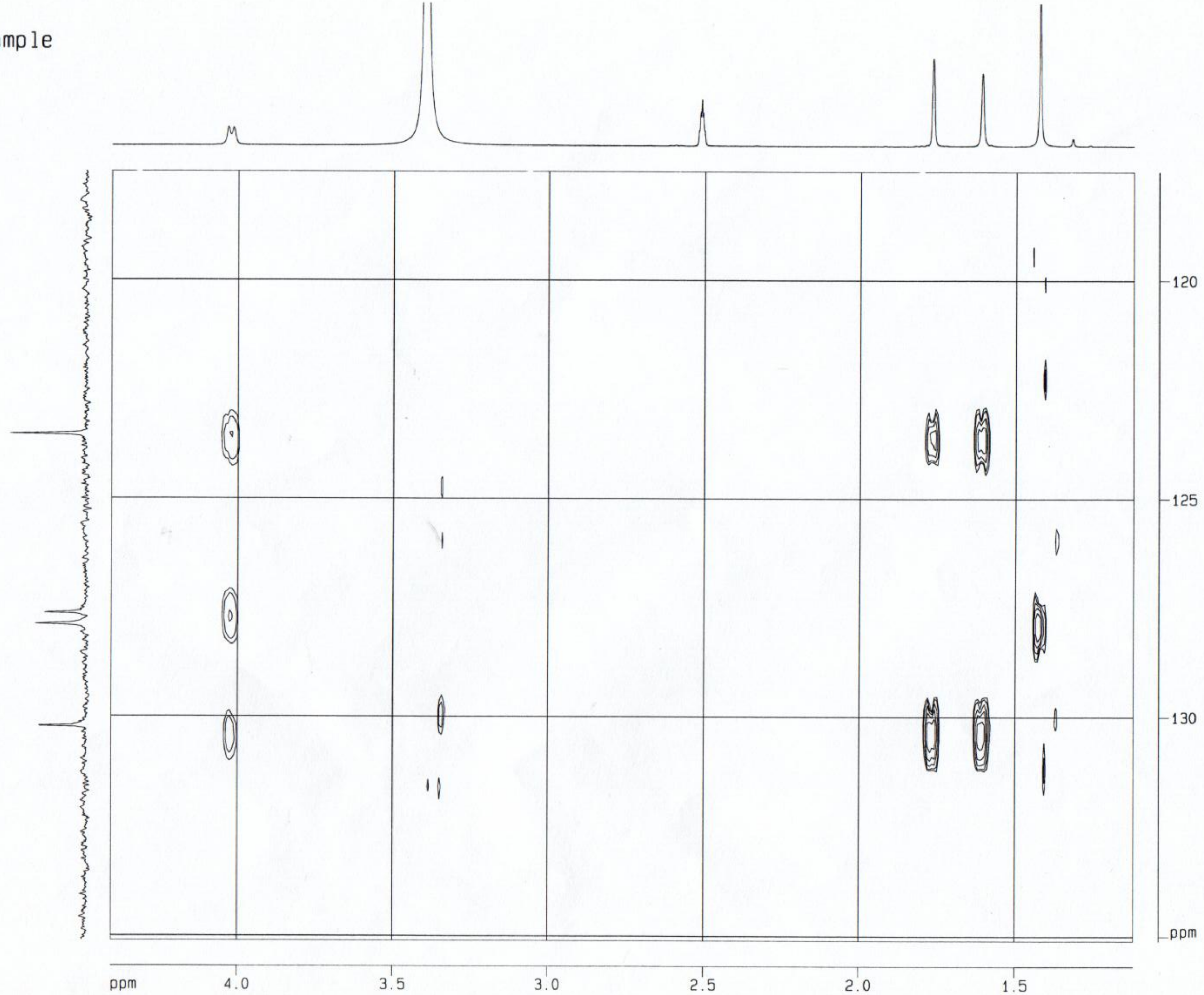

HMBC of sample
II-1-2

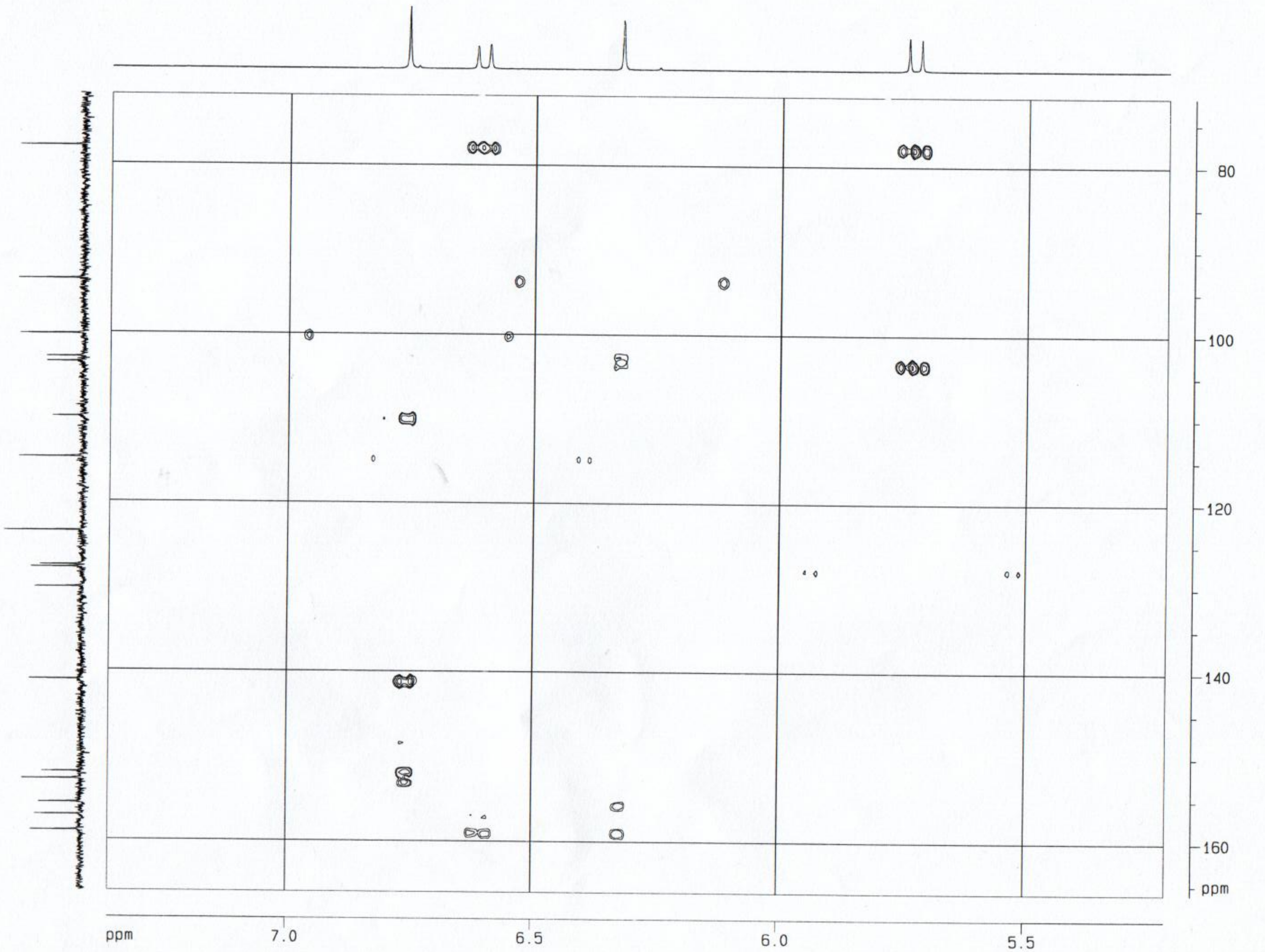

Table S1 Growth (%) of tumor cell PC12 treated with xanthones

compound	concentration (mg/ml)						
	0	1.25	2.5	5	10	20	40
**1**	100.00	97.81	88.67	96.34	76.18	59.43	41.79
**2**	100.00	102.56	100.00	102.05	92.05	70.77	65.77
**3**	100.00	102.37	98.14	96.60	94.42	76.28	73.53
**4**	100.00	105.48	108.30	108.59	104.54	93.14	63.06
**5**	100.00	105.38	112.15	113.88	99.75	77.63	49.88
**6**	100.00	100.40	108.00	96.35	84.10	100.69	61.43
**7**	100.00	102.83	99.56	92.03	88.42	92.77	101.56
**8**	100.00	93.01	90.08	97.95	95.75	87.49	73.41
**9**	100.00	108.13	108.62	106.68	96.01	91.77	111.18
**10**	100.00	109.73	106.39	109.98	102.15	108.83	103.14
**11**	100.00	91.97	91.75	95.30	86.66	60.15	33.93
**12**	100.00	95.88	103.30	94.33	93.79	92.00	85.04
**13**	100.00	107.50	98.80	110.60	96.60	96.60	86.50
**14**	100.00	100.00	92.30	100.20	96.00	95.80	95.70

Table S2 Growth (%) of tumor cell U87 treated with xanthones

compound	concentration (mg/ml)								
	0.00	0.31	0.63	1.25	2.50	5.00	10.00	20.00	40.00
**1**	100.00	88.03	87.25	75.56	52.07	40.04	29.45	/	/
**2**	100.00	/	/	116.90	113.55	108.06	76.70	36.92	32.75
**3**	100.00	/	/	105.10	117.21	104.82	77.85	44.41	34.91
**4**	100.00	/	/	114.04	114.49	82.13	54.01	39.85	37.72
**5**	100.00	/	/	93.28	90.91	68.47	86.57	47.70	38.95
**6**	100.00	/	/	156.60	151.18	134.77	138.26	96.40	62.41
**7**	100.00	/	/	110.32	103.62	99.37	108.10	96.63	70.03
**8**	100.00	/	/	89.48	81.26	85.41	101.13	61.87	52.20
**9**	100.00	/	/	103.79	77.15	79.20	88.96	53.77	48.77
**10**	100.00	/	/	84.20	71.34	78.27	61.79	51.11	48.33
**11**	100.00	87.81	88.58	88.58	94.43	68.94	37.73	/	/
**12**	100.00	93.12	92.99	89.24	94.54	82.96	57.80	/	/
**13**	100.00	97.92	88.70	88.85	89.57	88.74	78.04	/	/
**14**	100.00	91.47	74.36	76.74	83.39	68.90	60.58	/	/

Table S3. Linear regression data, LOD, and LOQ of the investigated compound **1**[a].

Regression equation	Test range (μg/kg)	R^2	LOD (μg/kg)	LOQ (μg/kg)	**1** (μg/kg, fresh)
Y=48.60X-1.38	0.12-1000	0.9967	0.08	0.50	13.89 ±0.57

[a] Values are means ±SD expressed in μg/kg fresh matter (n=3).
